# The Genome of the Human Pathogen *Candida albicans* Is Shaped by Mutation and Cryptic Sexual Recombination

**DOI:** 10.1128/mBio.01205-18

**Published:** 2018-09-18

**Authors:** Joshua M. Wang, Richard J. Bennett, Matthew Z. Anderson

**Affiliations:** a Department of Molecular Microbiology and Immunology, Brown University, Providence, Rhode Island, USA; b Department of Microbiology, The Ohio State University, Columbus, Ohio, USA; c Department of Microbial Infection and Immunity, The Ohio State University, Columbus, Ohio, USA; University of Minnesota Medical School

**Keywords:** *Candida*, evolution, loss of heteroxygosity, parasex, recombination

## Abstract

The opportunistic fungal pathogen Candida albicans lacks a conventional sexual program and is thought to evolve, at least primarily, through the clonal acquisition of genetic changes. Here, we performed an analysis of heterozygous diploid genomes from 21 clinical isolates to determine the natural evolutionary processes acting on the C. albicans genome. Mutation and recombination shaped the genomic landscape among the C. albicans isolates. Strain-specific single nucleotide polymorphisms (SNPs) and insertions/deletions (indels) clustered across the genome. Additionally, loss-of-heterozygosity (LOH) events contributed substantially to genotypic variation, with most long-tract LOH events extending to the ends of the chromosomes suggestive of repair via break-induced replication. Consistent with a model of inheritance by descent, most polymorphisms were shared between closely related strains. However, some isolates contained highly mosaic genomes consistent with strains having experienced interclade recombination during their evolutionary history. A detailed examination of mitochondrial genomes also revealed clear examples of interclade recombination among sequenced strains. These analyses therefore establish that both (para)sexual recombination and mitotic mutational processes drive evolution of this important pathogen. To further facilitate the study of C. albicans genomes, we also introduce an online platform, SNPMap, to examine SNP patterns in sequenced isolates.

## Abstract (precis)

Mutations introduce variation into the genome upon which selection can act. Defining the nature of these changes is critical for determining species evolution, as well as for understanding the genetic changes driving important cellular processes. The heterozygous diploid fungus Candida albicans is both a frequent commensal organism and a prevalent opportunistic pathogen. A prevailing theory is that C. albicans evolves primarily through the gradual buildup of mitotic mutations, and a pressing issue is whether sexual or parasexual processes also operate within natural populations. Here, we establish that the C. albicans genome evolves by a combination of localized mutation and both short-tract and long-tract loss-of-heterozygosity (LOH) events within the sequenced isolates. Mutations are more prevalent within noncoding and heterozygous regions and LOH increases towards chromosome ends. Furthermore, we provide evidence for genetic exchange between isolates, establishing that sexual or parasexual processes have contributed to the diversity of both nuclear and mitochondrial genomes.

## INTRODUCTION

A wide variety of genetic events contribute to the evolution of eukaryotic genomes. In asexual cells, haploid genomes evolve via the accumulation of point mutations as well as undergo recombination events that drive DNA expansions/contractions (indels). Heterozygous (het) diploid genomes also have the capacity to undergo loss-of-heterozygosity (LOH) events, in which genetic information is lost from one of the two chromosome (Chr) homologs. In addition, both haploid and diploid genomes may experience large-scale chromosomal changes such as gross rearrangements or acquisition of supernumerary chromosomes or other forms of aneuploidy (1, 2).

Many eukaryotic species also generate genetic diversity via sexual reproduction. Here, recombination between individuals provides an efficient mechanism for producing diverse progeny. Sexual reproduction can therefore promote adaptation to new environments more rapidly than asexual propagation (3, 4). However, associated fitness costs are incurred due to the energetic requirements of sex and to the fact that only 50% of parental alleles are passed on to sexual progeny (5–7). Sex can also be detrimental by breaking up highly beneficial allelic combinations (5, 8). Facultative sexuality, the ability to alternate between sexual and asexual forms of reproduction, promotes a more flexible lifestyle that can accelerate adaptation in response to environmental pressures (4, 9).

Candida albicans is a prevalent opportunistic fungal pathogen that was long thought to represent an obligate asexual species (10, 11). However, mating of diploid cells has been described in the laboratory and produces tetraploid cells that then return to the diploid state via a parasexual process of concerted chromosome loss (CCL) (12–15). Efficient mating requires that C. albicans cells undergo a phenotypic transition from the sterile “white” state to the mating-competent “opaque” state (16). Conjugation of opaque cells can occur via heterothallic or homothallic mating (17), and recombination during CCL involves Spo11, a conserved “meiosis-specific” factor involved in DNA double-strand-break formation across diverse eukaryotes (13, 18).

Clinical isolates of C. albicans exhibit a largely clonal population structure despite the potential for recombination via parasexual reproduction (19, 20). Multilocus sequence typing (MLST) separates C. albicans isolates into 17 clades, although discrepancies between MLST haplotypes and individual mutations suggested that recombination may act to generate new allelic variants (21). Analysis of a limited number of haploid mitochondrial loci also revealed allelic mixtures that suggest that recombination may have occurred within C. albicans populations (22, 23). However, despite these observations, C. albicans is still often described as an asexual species that does not undergo mating or recombination in nature (24, 25). Prior studies focused on a subset of genomic loci and presented conflicting evidence regarding the role of recombination in shaping C. albicans evolution (19, 21–23, 26), which can now be addressed by a detailed analysis of full-genome sequences.

In this work, we examined natural evolution of the diploid genome in the 21 sequenced C. albicans isolates that represent different clades, different sites of infection, and different countries of origin (27, 28). We reveal that emergent single nucleotide polymorphisms (SNPs) and indels often cluster together in the genome and occur more frequently in heterozygous (het) regions than in homozygous (hom) regions. Moreover, we highlight that both nuclear and mitochondrial genomes appear highly admixed in some isolates, which is indicative of genetic recombination having occurred between clades. Overall, our results establish that the C. albicans genome is a highly dynamic landscape shaped both by local mutations and by large-scale rearrangements and that sexual or parasexual mating has made a significant contribution to genotypic variation.

## RESULTS

**FIG 1:**
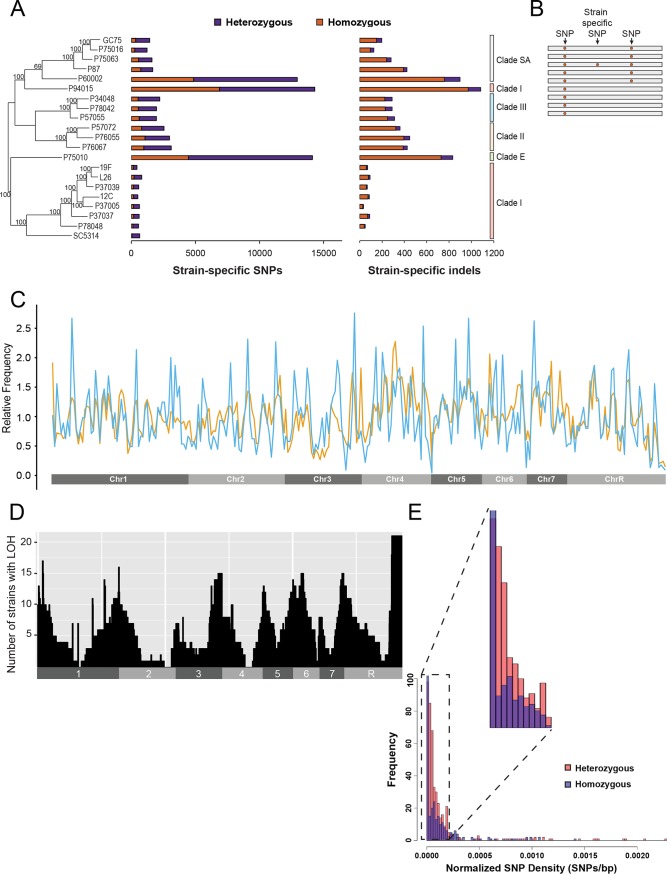
Distribution of polymorphisms among 21 clinical isolates of C. albicans. (A) The numbers of heterozygous (purple) and homozygous (orange) strain-specific SNPs and insertion/deletions (indels) are plotted for each isolate. Clade designations for each isolate are color coded (right), and bootstrap values are placed within the phylogenetic tree (left). (B) A cartoon representation depicts the two types of sequence variants among the set of 21 isolates: single nucleotide polymorphisms (SNPs) shared by multiple isolates and SNPs that are specific to individual strains. Variants harbored by multiple strains suggest origins in a common ancestor, whereas strain-specific polymorphisms likely arose specifically in individual strain backgrounds. (C) Relative frequencies of strain-specific SNPs (blue) and strain-specific indels (orange) summed across all strains were plotted across the genome using 5-kb sliding windows. (D) Number of strains that show LOH for 50-kb windows across the C. albicans genome aligned to their chromosomal position. (E) The SNP frequency for each of the contiguous 445 heterozygous (het) and 468 homozygous (hom) regions across the 21 sequenced genomes was plotted. Normalized SNP density was calculated using the number of SNPs within a het or hom region divided by the region length and was significantly elevated for het regions (red) relative to hom regions (blue).

The availability of whole-genome sequencing data for 21 diverse C. albicans isolates (27) provided an opportunity to determine how genetic diversity is generated between strains in nature. The C. albicans diploid genome is ∼14 Mb and consists of eight chromosomes harboring ∼6,100 genes (27, 29, 30). SNPs occur at a frequency of ∼0.3% (i.e., an average of 1 SNP every 330 bp) between chromosome homologs in the standard laboratory strain SC5314 (27, 31). Among the 21 isolates, we found that SNP frequencies ranged from 0.5% between closely related strains within clade I to 1.1% between strains from different clades (see Table S1 in the supplemental material). A previous phylogenetic reconstruction using 112,223 SNP positions found that most strains matched their previously assigned fingerprinting clades and MLST subtypes, with the exception of P94015, which clustered separately from other clade I strains (Fig. 1A) (27). The finding of strong bootstrap values across the constructed phylogeny of these strains supports the idea of a primarily clonal lifestyle in which most polymorphisms are consistent with inheritance by descent (Fig. 1B). Accordingly, SNPs and indels fit a nonrandom distribution across the 21 sequenced isolates χ^2^ ((SNPs; 20, N = 302641) = 83118, *P* < 2.2E–16, (indels; 20, N = 19581) = 13825, *P* < 2.2E–16) (see Fig. S1 in the supplemental material).

10.1128/mBio.01205-18.5 TABLE S1 Percentage of the genome harboring SNPs among the sequenced C. albicans isolates. Download Table S1, PDF file, 1 MB. Copyright © 2018 Wang et al. 2018 Wang et al. This content is distributed under the terms of the Creative Commons Attribution 4.0 International license.

10.1128/mBio.01205-18.2 FIG S1 The distribution of polymorphisms is nonrandom. The number of strains harboring each SNP (A) or indel (B) was determined and the frequency plotted. A best-fit line (red) was plotted for each distribution. Download FIG S1, PDF file, 0.8 MB. Copyright © 2018 Wang et al. 2018 Wang et al. This content is distributed under the terms of the Creative Commons Attribution 4.0 International license.

### Distribution of strain-specific polymorphisms across the *C. albicans* genome.

LOH events can distort the patterns of SNPs inherited from ancestral strains (Fig. S2). To help limit these confounding effects, we restricted most analyses to “strain-specific” SNPs and indels that are unique to individual strains in our collection (Fig. 1B). Approximately 25% of all SNP positions and 10% of all indel positions were strain specific (66,086 and 6,474 events, respectively; Tables S2 and S3).

10.1128/mBio.01205-18.6 TABLE S2 Strain-specific SNPs among sequenced C. albicans isolates. Download Table S2, XLSX file, 1.9 MB. Copyright © 2018 Wang et al. 2018 Wang et al. This content is distributed under the terms of the Creative Commons Attribution 4.0 International license.

10.1128/mBio.01205-18.7 TABLE S3 Strain-specific indels among sequenced C. albicans isolates. Download Table S3, CSV file, 0.2 MB. Copyright © 2018 Wang et al. 2018 Wang et al. This content is distributed under the terms of the Creative Commons Attribution 4.0 International license.

10.1128/mBio.01205-18.3 FIG S2 LOH can obfuscate patterns of inheritance by descent of shared polymorphisms. LOH of opposing alleles in a common ancestor can make it appear that mutations arose independently despite the mutations sharing a common origin. LOH of homolog A in clade III produces a SNP pattern different from that of clade II although t arose from the same ancestral strain. Download FIG S2, PDF file, 0.8 MB. Copyright © 2018 Wang et al. 2018 Wang et al. This content is distributed under the terms of the Creative Commons Attribution 4.0 International license.

Analysis of the genome-wide distribution of strain-specific SNPs and indels across the 21 genomes revealed that these mutation types showed significant clustering with one another (Pearson, *t* = 11.64, df = 286, *P*  < 2.2E−16; Fig. 1C). Multiple SNPs often occurred within 100 bp of an indel (Fig. S3A), as was confirmed via Sanger sequencing of selected regions (see Fig. S4 at https://drive.google.com/drive/u/0/folders/1u0LiWcB-yufoL4iQOn_bQQ2ercxB-F6R). Enrichment of SNPs was observed immediately adjacent to indels (within 10 bp) but not within indels [Wilcoxon test, W(1.79E7), *P*  < 2.2E−16] (Fig. S3B). Three strains, P60002, P75010, and P94015, carried genes that encoded a large proportion of strain-specific mutations reflective of their longer branch lengths in the phylogenetic tree (Fig. 1A), which could potentially skew the analysis. However, even after removing these three strains from the analysis and reducing the four major clades to three representative strains each, we still observed a significant association between SNPs and indels [Wilcoxon test, W(3.04E7), *P*  < 2.2E-16] (see Fig. S5A at https://drive.google.com/drive/u/0/folders/1u0LiWcB-yufoL4iQOn_bQQ2ercxB-F6R). This association highlights that these mutagenic events often occur in close proximity to one another, as has been observed in other eukaryotic species (32), and that indel formation or the associated DNA repair processes may be mutagenic in C. albicans.

10.1128/mBio.01205-18.4 FIG S3 Unique SNPs and unique indels. Download FIG S3, PDF file, 1.2 MB. Copyright © 2018 Wang et al. 2018 Wang et al. This content is distributed under the terms of the Creative Commons Attribution 4.0 International license.

### Association between LOH recombination events and base substitution mutations.

We next examined the global distribution of LOH tracts across the 21 genomes. LOH tracts were defined as contiguous 5-kb windows with a high frequency of homozygous SNPs (>0.4 events/kb; see Materials and Methods and reference 27). Plotting the incidence of LOH for all chromosomes revealed a clear pattern whereby the prevalence of LOH increased along each chromosome arm in progressing from centromere to telomere (Fig. 1D; see also Fig. S6 at https://drive.google.com/drive/u/0/folders/1u0LiWcB-yufoL4iQOn_bQQ2ercxB-F6R). In fact, the overwhelming majority of all long-tract LOH regions (155 of 170 regions larger than 50 kb) extended to the ends of the corresponding chromosomes (Table S4), indicating that most large LOH events initiate on one arm and then proceed to the end of the chromosome.

10.1128/mBio.01205-18.8 TABLE S4 Heterozygous and homozygous regions for the sequenced C. albicans isolates. Download Table S4, XLSX file, 0.3 MB. Copyright © 2018 Wang et al. 2018 Wang et al. This content is distributed under the terms of the Creative Commons Attribution 4.0 International license.

Several studies have shown that the underlying genomic context can impact mutation rates, including the observation that mutation rates were higher in heterozygotes than in homozygotes during meiosis (33). We therefore examined mutational patterns in the 21 C. albicans genomes that consist of a mosaic of heterozygous and homozygous regions. We subdivided C. albicans genomes into heterozygous (het) and homozygous (hom) regions using defined criteria for all SNPs (see Materials and Methods), resulting in 468 het and 445 hom regions, respectively (Table S4). The het regions covered a total of 71.1% of the genome and hom regions 28.9%; het tracts were therefore considerably longer on average than hom tracts (∼480,000 bp versus 186,000 bp, respectively). Definition of het and hom regions using all SNPs allowed examination of the frequencies of strain-specific SNPs within these regions. Strain-specific SNPs comprised only 3% of all SNPs within these genomes and therefore did not contribute substantially to the designation of het and hom regions. Using these designations, het regions contained significantly higher densities of strain-specific SNPs than hom regions (1.4E−4 SNPs/bp versus 7.3E−5 SNPs/bp, respectively; Brunner-Munzel [BM] test value = −10.6, df = 786.6, *P*  < 2E−16; Fig. 1E). Again, to determine if the three outlier strains, P60002, P75010, and P94015, biased this analysis, we removed these three strains and reduced the four major clades to three representative strains each, and yet there were still significantly higher densities of strain-specific SNPs within het regions than within hom regions (BM test = −7.558, df = 377.0, *P* = 3.14E−13). Furthermore, all 21 isolates exhibited the same bias toward het regions containing more strain-specific SNPs than hom regions (two-tailed BM test = −1.11, df = 38.5, *P* = 0.28). This indicates that mutations preferentially accumulate in het regions rather than hom regions during natural evolution of C. albicans isolates, as was seen for het regions versus hom regions during meiotic propagation (33).

### Identity by descent during *C. albicans* evolution.

**FIG 2:**
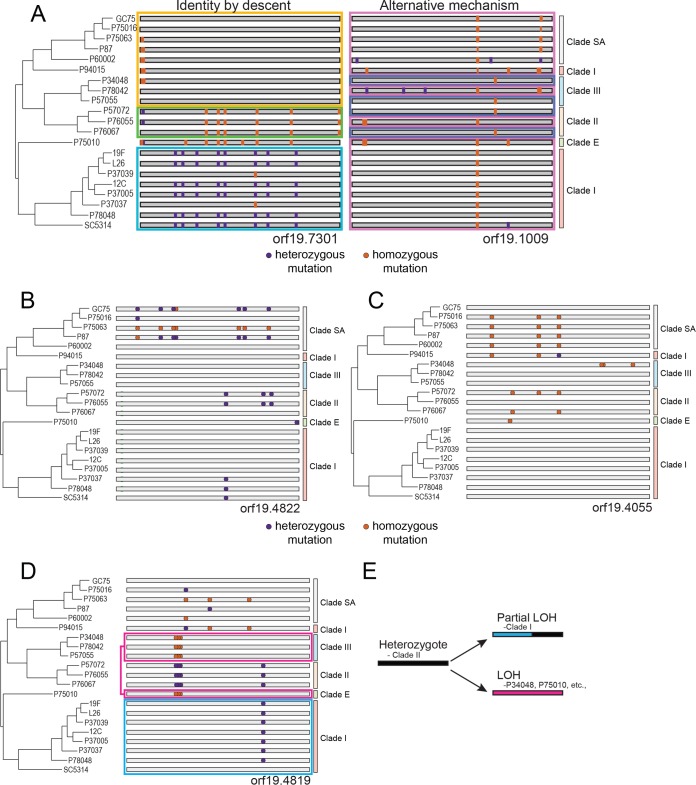
Mutational patterns following identity by descent and loss of heterozygosity are widespread. (A) Two patterns of polymorphisms exist within sequenced genomes. One pattern (depicted on the left) shows polymorphisms that are phylogenetically congruent (indicative of identity by descent), and the other (depicted on the right) shows polymorphisms that violate the phylogenetic relationship of the strains based on all variant positions (indicative of mechanisms other than direct inheritance). Shading denotes similarity in overall SNP patterns grouped by color. Heterozygous SNPs are purple, and homozygous SNPs are orange. (B and C) Polymorphisms for two loci (orf19.4822 [B] and orf19.4055 [C]) that display SNP patterns consistent with inheritance by descent across the sequenced C. albicans genomes are plotted. (D) The polymorphism pattern is plotted for a locus that does not follow inheritance by descent. Identical genotypes are color coded and connected to each other. (E) A cartoon depicting LOH of heterozygous positions in opposing directions provides the most parsimonious explanation for the observed SNP patterns across the C. albicans phylogeny.

A hallmark of phylogenetic reconstructions in asexual species is the ability to track the relatedness of isolates based on inherited polymorphisms (21, 34, 35). Reconstruction often relies on a maximum parsimony model of “identity by descent,” in which more closely related strains share a greater percentage of shared polymorphisms (Fig. 2A). C. albicans SNP patterns generally follow this model as evidenced by strong bootstrap support at almost all nodes of the phylogenetic tree and visual examination of SNP patterns (Fig. 2B and C; see also Fig. S7 at https://drive.google.com/drive/u/0/folders/1u0LiWcB-yufoL4iQOn_bQQ2ercxB-F6R). Strikingly, however, certain regions of the genome exhibited clear violations of identity by descent (Fig. 2D and E; see also Fig. S8 at https://drive.google.com/drive/u/0/folders/1u0LiWcB-yufoL4iQOn_bQQ2ercxB-F6R). In heterozygous diploid genomes, these deviations could potentially arise through two mechanisms: (i) through sexual recombination between genetically distinct isolates or (ii) through multiple independent LOH events that obfuscate the actual pattern of descent (see Fig. S9 at https://drive.google.com/drive/u/0/folders/1u0LiWcB-yufoL4iQOn_bQQ2ercxB-F6R). In the latter case, multiple LOH events could cause loss or retention of SNPs through homozygosis of one chromosome homolog or the other, thereby generating a subset of isolates that appeared “recombinant,” i.e., appeared to have intermixed genetic content from two different relatives. Such a history can sometimes be inferred by a comparison of SNP patterns within the region of interest in multiple extant strains (Fig. 2A).

We note that certain regions in the C. albicans genome are consistent with divergent short-tract LOH events having occurred during evolution (Fig. 2D and E; see also Fig. S8 at https://drive.google.com/drive/u/0/folders/1u0LiWcB-yufoL4iQOn_bQQ2ercxB-F6R and the supplemental material). In line with this, we identified 514 nonoverlapping 25-kb windows across the 21 sequenced isolates that do not harbor similar polymorphisms to closely related strains and that could represent regions that had experienced LOH. Interestingly, two strains, P60002 and P94015, contained 390 (75.9%) of these regions, although only 214 (54.9%) of the 390 incongruent regions in these two strains overlapped with LOH tracts (hom regions) in these isolates. In contrast, the majority (117 of 124) of the incongruent regions in all other strains overlapped with LOH tracts. This suggests that incongruence in polymorphisms in most strains likely results from divergent LOH events but that LOH cannot easily explain the majority of incongruent polymorphic patterns observed in P60002 and P94015.

### Evidence for recombination in natural isolates of *C. albicans*.

Previous studies provided conflicting messages regarding recombination in natural populations of C. albicans (19, 21–23, 26), and none examined whole-genome data for evidence of interclade mixing. We therefore examined the 21 sequenced genomes for mixed evolutionary histories. The similarity of genomic segments from each strain to the overall phylogenetic tree was determined by analysis of SNP patterns using 25-kb sliding windows. To aid visualization of SNP patterns, we developed a custom tool, SNPMap (http://snpmap.asc.ohio-state.edu/), which allows users to map the positions of individual mutations, mutation types, and het/hom tracts across user-defined regions of the 21 genomes.

**FIG 3:**
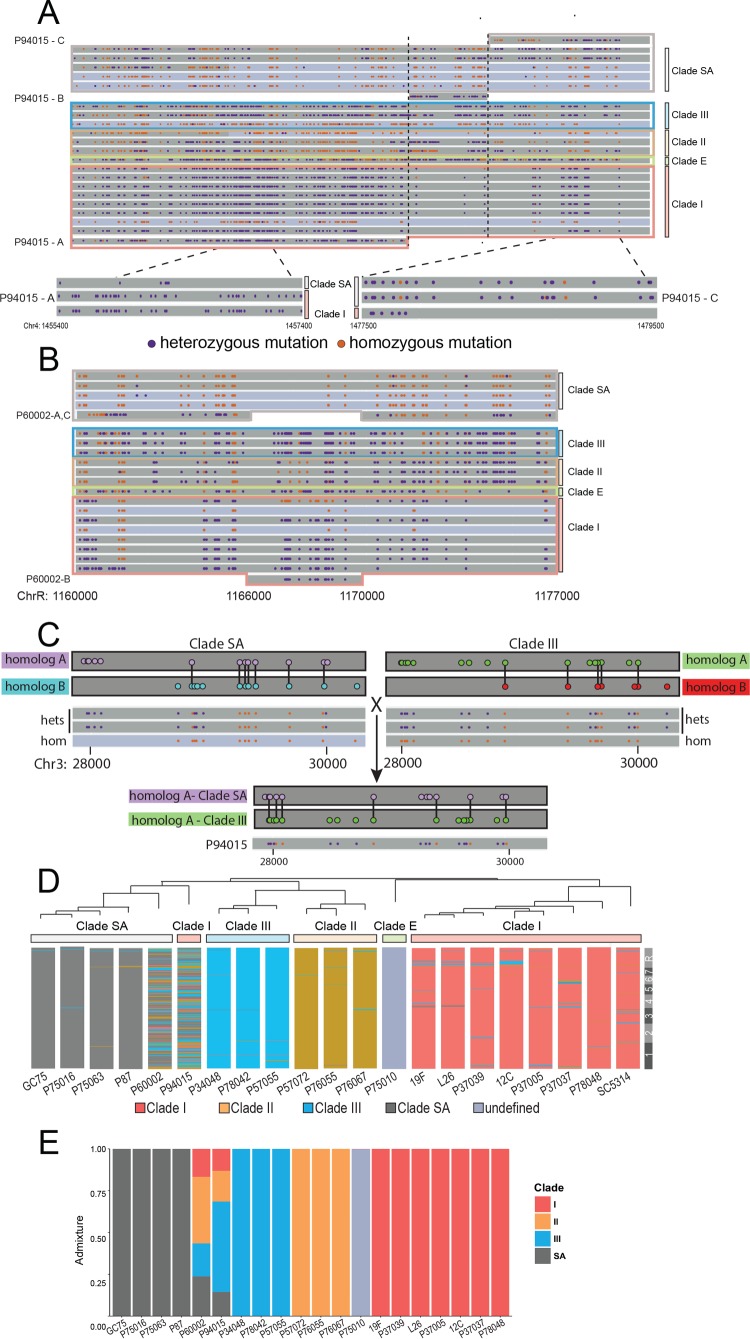
Evidence for recombination in C. albicans isolates. (A) All SNPs are shown for a 20-kb region of Chr4 for the 21 sequenced genomes. For each strain, dark gray bars indicate heterozygous genomic regions and light blue bars indicate regions that are mostly homozygous. SNPs that are heterozygous and homozygous compared to SC5314 are purple and orange, respectively. The SNP pattern in P94015 is highlighted to indicate one region with homology to clade I (P94015-A; outlined in red) next to a region without clear homology to any specific clade (P94015-B) followed by a region with homology to clade SA (P94015-C; outlined in gray). Zoomed segments from P94015-A and P94015-C showing identity to individual SNPs from clades I and SA, respectively, are displayed at the bottom of the figure panels. Clade designations for each isolate are color coded. All SNPs that are at the same position involved the same base substitutions. (B) The SNP patterns for an ∼20-kb region of ChrR highlights recombinant SNP patterns for P60002 and is labeled as described for panel A. The DNA segments corresponding to different clades are aligned next to the appropriate group and labeled according to homologous tracts. (C) SNPs for a 2.5-kb region of Chr3 were phased by comparison of clade SA and III strains that were heterozygous or homozygous for this region. Homozygous SNPs are connected across homologs by a black line. The SNP pattern of this region in P94015 matches the product of hybridization between the panel A homologs from clade SA and clade III (both the position of the SNP and the actual base substitution matched). Cartoons of phased SNP homologs are represented above the collective SNP pattern for each strain. Heterozygous and homozygous SNPs are purple and orange in the genome snapshots underneath the phased homolog cartoons, respectively. (D) Consensus SNP patterns for each clade were determined and used to assess similarities of 25-kb sliding windows in each isolate to the clade consensus patterns. The closest match for each window was color coded by clade. Two strains, P60002 and P94015, contained a large portion of regions assigned to multiple clades compared to all other strains. Dark gray, SA; blue, clade III; mustard, clade II; red, clade I; light gray, no clade consensus. (E) Each strain was compared to a consensus SNP pattern for the major represented clades using four K-clusters. The fraction of the genome corresponding to each of the four clusters (dark gray, SA; blue, clade III; mustard, clade II; red, clade I) is represented within the bar height for each strain. Light gray denotes an outlier clade.

Our analysis focused on P60002 and P94015, two isolates that had the weakest bootstrap support within the C. albicans phylogeny and that clustered with different strains by MLST or DNA fingerprinting analysis (27). The latter data could be consistent with their genomes being formed by recombination between isolates. In support of this, examination of Chr4 in P94015 identified one region with clear homology to clade I in close proximity to a region homologous to clade SA (Fig. 3A; see also Table S5). The region with homology to clade I (labeled P94015-A in Fig. 3A) shares a large number of SNPs that are present throughout clade I but absent in all other strains with the exception of P94015 (Table S5). Next to this region, a 1-kb segment (region P94015-B) was seen that lacks clear homology to any of the other sequenced isolates while, adjacent to this, the SNP pattern in P94015 was virtually identical to those of two clade SA isolates (region P94015-C). We similarly identified a region on ChrR in P60002 that switched homology between clades I and SA (Fig. 3B). A ∼4-kb stretch of the genome (region P60002-B; ChrR:1166000.1170000) harbored heterozygous SNPs that perfectly matched clade I SNPs and was flanked by segments that matched the SNP pattern among clade SA isolates (region P60002-A,C). These regions could therefore have been shaped by recombination between isolates.

10.1128/mBio.01205-18.9 TABLE S5 SNPs across the highlighted recombinant regions for P94015. Download Table S5, XLSX file, 0.01 MB. Copyright © 2018 Wang et al. 2018 Wang et al. This content is distributed under the terms of the Creative Commons Attribution 4.0 International license.

10.1128/mBio.01205-18.10 TABLE S6 Genes with significant enrichment of strain-specific SNPs. Lists of genes at three different levels of SNPs per nucleotide cutoff are provided. Download Table S6, XLSX file, 0.02 MB. Copyright © 2018 Wang et al. 2018 Wang et al. This content is distributed under the terms of the Creative Commons Attribution 4.0 International license.

Mating between isolates from different clades would be expected to generate hybrid DNA regions, with SNPs on one homolog of the recombinant strain matching those in one clade and SNPs on the other homolog matching those in a second clade. Identifying inherited SNPs following C. albicans mating in nature is complicated by the fact that, with the exception of SC5314 (31), haplotypes are not available for the 21 C. albicans genomes. Despite this, phasing of heterozygous SNPs for some isolates can be inferred using SNP patterns from related strains that have undergone LOH for that region (Fig. 3C). The region that experienced LOH would retain only the SNPs that reside on the same homolog (i.e., those that are phased). Using this approach, we phased SNPs for chromosomal regions that are heterozygous in some isolates but homozygous in closely related strains. The existence of multiple isolates that have undergone LOH for either of the two homologs strengthens the confidence of phasing assignments within a given clade.

We applied this approach to a region on Chr3 in P94105 that contains polymorphisms identical both to those on homolog A of a clade SA strain (12 of 12 SNPs are identical) and to those on homolog A from a clade III isolate (15 of 15 SNPs are identical) (Fig. 3C). Both the SNP positions and the nucleotide identities are conserved across this region in P94105 compared to the corresponding homologs from clade SA and clade III isolates. The identification of this hybrid region therefore provides a striking example of P94105 inheriting one homolog from a clade SA strain and one homolog from a clade III strain and establishes a nonclonal origin for this isolate.

To examine the global patterns of admixing among the set of 21 isolates, the distribution of all variant positions in each strain was compared to the consensus pattern for each clade using sliding 25-kb windows. The SNP patterns of most isolates resembled the consensus pattern for their assigned clade (98.5% of genomic windows matched their assigned clade), as expected for a population propagating clonally (Fig. 3D). In contrast, many regions within the P60002 and P94015 genomes showed homology to multiple different clades, producing highly mosaic genomes (Fig. 3D). Here, the genomes of P60002 and P94015 matched their assigned clades for only 58.3% and 76.7% of sliding windows, respectively (*P* = 1.14E−10). The majority of genomic regions in P94015 aligned with clade I (the genome is mostly color coded red in Fig. 3D), whereas numerous segments aligned to regions from three other major clades (SA, II, and III). In the case of P60002, clade SA regions made up the majority of the genome, with a smaller number of regions matching clade I or, to a lesser extent, clade II. In line with this, the branch point leading to P60002 is the least well-supported node in the phylogenetic reconstruction of all 21 isolates (27).

C. albicans strains containing mosaic genomes should display evidence of classical admixture and allow an estimation of clade contributions to the mosaicism. To assess admixture, we restricted analysis to the four major clades represented here and assigned the fraction of SNPs within each genome to each of the clades. Consistent with the previous sliding approach to assign sequence homology, P60002 and P94015 were identified as the only strains with significant levels of admixture (Fig. 3E). Both strains displayed genetic contributions from all four major clades, suggesting that relatively ancient recombination events generated these admixed genomes. The most parsimonious explanation for these highly mosaic genomes is that they are the products of mating and recombination between isolates from multiple C. albicans clades.

### Analysis of mitochondrial genomes in *C. albicans* isolates.

Haploid mitochondrial genomes provide a more simplified context for the search for evidence of recombination than heterozygous diploid genomes. In Saccharomyces cerevisiae, mitochondrial genomes are biparentally inherited following mating, and recombination between parental genomes can occur prior to zygote division (36). We therefore performed the first comparative analysis of global SNP patterns in C. albicans mitochondrial genomes using sequencing data from the set of 21 isolates. The mitochondrial genome in C. albicans is ∼41 kb in size, and a total of 1,847 SNPs (and 0 indels) were annotated within the 21 isolates, with an average SNP density of 1 polymorphism every 476 bp. The mitochondrial genome was highly heterogeneous and included areas of high SNP density (e.g., ChrM positions 15000 to 20000) and regions devoid of polymorphisms (e.g., ChrM: 6000–12000). Furthermore, of the 1,847 annotated mitochondrial SNPs, only 39 were strain specific in the set of 21 sequenced genomes (Table S2).

**FIG 4:**
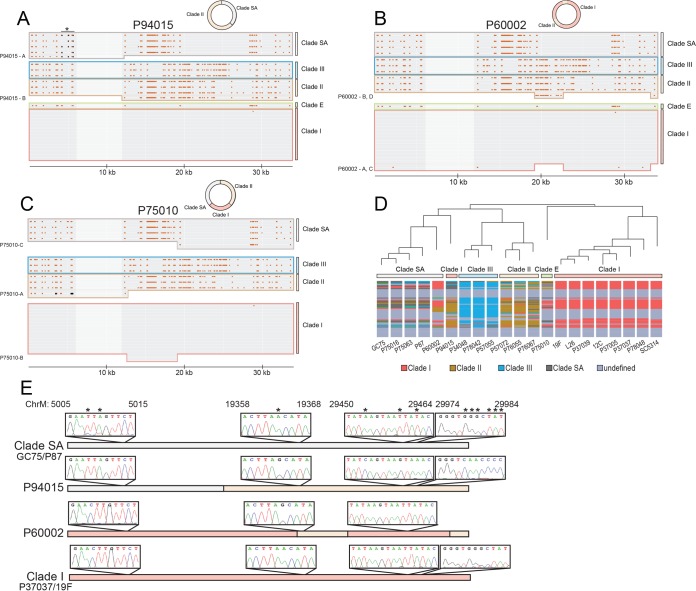
Mitochondrial genomes in C. albicans display recombinant genotypes. The mitochondrial (mt) genome sequences of 21 clinical isolates of C. albicans were compared. The positions of SNPs that differ from the SC5314 assembly are shown (excluding ChrM: 35000–41000 due to the absence of any SNPs in this region). (A to C) The mt genomes for P94015 (**A**), P60002 (**B**), and P75010 (**C**) are highlighted to show the mosaic configuration of SNPs relative to other clades for these strains. The mt genome in P94015 contains regions that share polymorphisms that are identical with those of clade SA (P94015-A) and clade II (P94015-B). A key region matching clade SA is marked with an asterisk, with the three clade SA-specific SNPs marked in black. In contrast, the P60002 mt genome aligns with sequences for clades I (P60002-A and P60002-C) and II (P60002-B and P60002-D). The mt genome of P75010 is homologous to clades II (P75010-A), I (P75010-B), and SA (P75010-C). Key clade II-specific SNPs in the P75010 alignment are marked in black. A 6-kb region devoid of SNPs is more lightly shaded. (D) The similarity of the mt genome from each isolate to consensus SNP patterns was determined for each clade in 2-kb sliding windows. The window was color coded to designate the clade with greatest similarity. Dark gray, SA; blue, clade III; mustard, clade II; red, clade I; light gray, no clade consensus. (E) Two strains from clade SA and clade I, along with P60002 and P94015, were subjected to Sanger sequencing across three separate 1-kb regions of their mitochondrial genomes. Chromatograms highlight variant positions in P60002 and P94015 consistent with recombination between clades as determined by genome analysis.

C. albicans mitochondrial genomes generally showed clade-specific SNP patterns that were again consistent with a clonal population structure, although the resolution of the SNP patterns was low due to relatively few clade-defining mitochondrial SNPs (Fig. 4). As with nuclear genomes, examination of mitochondrial genomes of P60002 and P94015 showed evidence of interclade mixing. For example, the mitochondrial genome of P94015 contained regions that aligned with mitochondrial segments from both clade SA and clade II (Fig. 4A). There, three polymorphisms were clade SA specific on ChrM: 1–6000 (region P94015-A; black dots), and all three are present in P94015 (Fig. 4A). An additional 2 of the 15 polymorphisms in region P94015-A are specific to this strain (Table S2). The remainder of the mitochondrial genome in P94015 (region P94015-B) harbors 144 polymorphisms matching the SNP pattern found in clade II (with the exception of two strain-specific SNPs). Recombination between clades I and II was even clearer in the mitochondrial genome of P60002, as the majority of this genome was identical to that of clade I (P60002 regions A and C), but a 4-kb segment (ChrM: 19000–22000) harbored 34 polymorphisms that were identical to clade II polymorphisms and were entirely absent in clade I (P60002 region B) (Fig. 4B; see also Fig. S10 at https://drive.google.com/drive/u/0/folders/1u0LiWcB-yufoL4iQOn_bQQ2ercxB-F6R). We also found that maximum parsimony approaches mostly separated the P90145 mitochondrial genome into clade II and SA regions and the P60002 mitochondrial genome into clade I and II regions (Fig. 4D), consistent with visual alignments (Fig. 4A and B). Results of direct Sanger sequencing of the mitochondrial genome (regions 5000 to 5700, 18500 to 19500, and 29000 to 30000) supported the SNP designations from whole-genome sequencing and therefore established that interclade recombination has occurred within the mitochondrial genomes of P60002 and P90145 (Fig. 4E; see also Fig. S11 at https://drive.google.com/drive/u/0/folders/1u0LiWcB-yufoL4iQOn_bQQ2ercxB-F6R).

We further note that the single representative of clade E in our collection, strain P75010, also displays strong evidence of recombination in its mitochondrial genome (Fig. 4C). The first ∼12 kb of P75010 (region P75010-A) aligns closely with clade II and harbors all three clade II-specific SNPs in this region (region P75010-A; black dots). The ∼7-kb region of P75010 (region P75010-B) that follows matches that of clade I strains and is followed by a region with clear homology to clade SA, harboring 22 of 25 clade SA-specific SNPs. Identity to several clades implies that multiple recombination events gave rise to this complex SNP pattern. Taken together, these results reveal that recombination events have occurred within C. albicans mitochondrial genomes and provide clear evidence that sexual/parasexual processes have occurred during C. albicans evolution.

## DISCUSSION

Our analysis of the C. albicans genome structure reveals a number of important aspects concerning mutational patterns occurring during the natural evolution of the species. We highlight that (i) there is a significant association between the positions of emergent SNPs and indels; (ii) diverse LOH events contribute to genetic inheritance, including long-tract LOH events that extend to the ends of the chromosomes; (iii) heterozygous regions of the genome accumulate more mutations than coding and homozygous regions; (iv) a subset of strains exhibit mosaic nuclear and mitochondrial genomes; and (v) analysis of specific chromosomal regions reveals clear evidence for interclade recombination.

### Mutations driving natural evolution of the *C. albicans* genome.

Mutation rates differ across eukaryotic genomes in a context-dependent manner (37, 38). We found that mutation patterns arising in natural C. albicans populations similarly exhibit a nonrandom distribution across the genome. Our analysis focused on the distribution of strain-specific SNPs, as these SNPs are likely to have emerged since these strains diverged from one another. We found that the location of strain-specific SNPs was biased toward heterozygous rather than homozygous regions of the genome. This is consistent with recent studies that showed that mutations arising during meiosis occurred more frequently within heterozygous regions of eukaryotic genomes than within homozygous regions, although the mechanism driving this bias is unknown (39, 40). Our results extend these findings by indicating preferential accumulation of mutations within heterozygous regions during mitotic growth in C. albicans, suggesting that elevated mutation rates may be a common feature associated with heterozygosity.

Our analysis also reveals that emergent SNPs and indels cluster together within the C. albicans genome, with a significant enrichment of SNPs within 10 bp of emergent indels (Fig S3). This is similar to what has been observed in other eukaryotic species where indels were found to promote elevated substitution rates close to the founding indel (32, 41–44).

### Evolutionary impact of loss of heterozygosity.

Loss-of-heterozygosity events are a frequent occurrence in C. albicans genomes (1, 45). Previous analysis of the 21 C. albicans genomes noted that LOH tracts can be highly variable in size, with several isolates having experienced whole-chromosome LOH (27). Here, we made the observation that the vast majority of very large LOH tracts (defined as segmental LOH tracts of >50 kb) initiated between the centromere and the telomere and extended to the chromosome ends. Such LOH events are likely due to break-induced replication, in which one chromosome homolog is used as a template to repair a double-strand DNA break in the other homolog, although reciprocal crossover events can also generate long LOH tracts (25, 46, 47). The breakpoints for most of these long-tract LOH events differed between individual strains, suggesting that independent LOH events had occurred. Short-tract LOH (<50 kb) was also common, with approximately half of these events being shared between related strains (see Table S4 in the supplemental material; see also Fig. S6 at https://drive.google.com/drive/u/0/folders/1u0LiWcB-yufoL4iQOn_bQQ2ercxB-F6R). Homozygosis of certain regions may confer a selective advantage given that LOH has been shown to alter C. albicans traits, from growth rates to virulence to drug resistance (28, 48–50).

The cumulative effects of all of these mutational forces on C. albicans genomes result in accelerated evolution of heterozygous regions relative to homozygous regions. Eventually, the rapid accumulation of deleterious mutations during clonal growth would be expected to result in a fitness decline due to Muller’s ratchet (51, 52). The occurrence of LOH may counterbalance these forces both by culling mutations from the genome and by reducing heterozygosity to low evolutionary rates. LOH events appear at different points during the evolution of individual strains, with a number of LOH tracts differing even between closely related strains. Indeed, the accumulation of strain-specific SNPs within more-ancestral LOH tracts demonstrates that these LOH events are not recent occurrences and additional mutations have emerged since their origin.

### Evidence for genetic exchange in clinical *C. albicans* isolates.

Studies of C. albicans population structure point to a largely clonal mode of reproduction, and yet there is also evidence of mixed evolutionary histories indicative of a species reproducing sexually/parasexually (20, 21, 23, 26, 53–55). Furthermore, genes harbored at the mating locus show evidence for ongoing selection consistent with a conserved role in regulating sexual/parasexual reproduction (56). In this study, we interrogated whole-genome data for evidence of genetic admixture, while noting that LOH events can complicate analysis of recombination (Fig. 2; see also Fig. S8 at https://drive.google.com/drive/u/0/folders/1u0LiWcB-yufoL4iQOn_bQQ2ercxB-F6R). We reveal that a subset of isolates contained mosaic genomes, consistent with those genomes being the products of mating between different C. albicans clades. Both nuclear and mitochondrial genomes of P60002 and P94015 show recombinant genotypes, supporting the idea of a (para)sexual origin for these strains. Genetic information from multiple clades contributed to these genomes, and recombination tracts ranged in length from a few kilobases to hundreds of kilobases (Fig. 3 and 4). The existence of a subset of C. albicans strains with mosaic genomes is similar to what has been observed in wild and domesticated strains of S. cerevisiae, where both nonmosaic and recombinant mosaic genomes have been identified (57). Analysis of admixed genomes in P60002 and P94015 suggests that recombination events may be relatively ancient, as recombination involves multiple clades and more-recent mutational events have obscured the precise evolutionary histories of these strains.

Disagreement between strain phylogeny data determined by MLST and Ca3 fingerprinting may also be indicative of recombination in the population. On the basis of Ca3 fingerprinting, P94015 should cluster with other clade I strains and is supported by MLST analysis, which groups P94015 and other MLST 6 strains closer to MLST 1/clade I than other groups (27). And yet, whole-genome sequencing clusters P94015 with clade SA rather than with other clade I strains. This could reflect how recombination has distorted the phylogenetic relationship between strains on the basis of analysis of a small subset of loci. Analysis of additional C. albicans isolates will help define the prevalence of recombination in the species and whether these recombination events are ancient or are more recent occurrences.

Critically, we identified regions in C. albicans genomes that exactly matched the pattern of recombinant SNPs expected from mating events occurring between two extant clades. This was exemplified by one region in P94015, which consisted of multiple SNPs that exactly matched those present in clade I and that were followed, after a short gap, by a run of SNPs that precisely matched those in clade SA (Fig. 3A). Moreover, recombination events were clearly evident in the mitochondrial genomes of at least 3 isolates (P60002, P75010, and P94015) among the 21 examined. Taken together, these studies provide the clearest evidence to date that C. albicans populations have been shaped by (para)sexual exchange.

In summary, C. albicans genomes reveal multiple signatures of the forces that have shaped genetic diversity within the species. Both short and long LOH events have played a major role in increasing population diversity, with large tracts extending to chromosome termini that impact hundreds to thousands of polymorphisms. Base substitution mutations and indels cluster within heterozygous regions of the genome, suggestive of faster evolution of these regions. Finally, recombination between isolates has generated mosaic nuclear and mitochondrial genomes, which could have enabled adaptation in the species. The diploid heterozygous genome of C. albicans is therefore a highly dynamic platform on which selection can act.

## MATERIALS AND METHODS

### Variant calling, processing, and display.

Whole-genome sequencing, variant identification, and loss-of-heterozygosity (LOH) windows were identified in a previous study (27). Briefly, BWA 0.5.9 (58) read alignments were filtered with a minimum mapping quality of 30 using SAMtools (59). To reduce the incidence of false-positive SNPs called near indels, poorly aligned regions were realigned using GATK RealignerTargetCreator and IndelRealigner (GATK version 1.4-14) (60). Both prior SNP variants and variants present in the mitochondrial genomes and indels for both the mitochondrial and nuclear genomes described here used GATK UnifiedGenotyper, and results were filtered using GATK VariantFiltration and hard filters (QD, <2.0; MQ, <40.0; FS,  >60.0; MQRankSum, less than 12.5; ReadPosRankSum, less than −8.0). SNPs were called homozygous if greater than 90% of the reads contained the nonreference nucleotide. This high threshold also reduced miscalling due to trisomic chromosomes/chromosomal regions.

To assess heterozygous and homozygous variant calls, the number of reads at each variant position was divided by the total number of reads at that position in that strain. On average, each variant position had 52.17 reads, with an interquartile range of 33 to 71 reads. The distribution of the allelic ratio at each variant is plotted in Fig. S12 at https://drive.google.com/drive/u/0/folders/1u0LiWcB-yufoL4iQOn_bQQ2ercxB-F6R. Dividing the mean number of reads of the allele by the total number of reads resulted in a value of 0.4499 for heterozygous positions and a value of 0.9889 for homozygous regions. Thus, a 90% threshold was used for homozygous regions whereas heterozygous regions span from 10 to 50%.

From this data set, strain-specific SNPs and indels were parsed into a separate set for additional analysis. Strain-specific variant features were required to be uniquely called in only 1 of the 21 strains at its genomic position. The data sets are available online in a searchable interface, using R shiny for the backend (https://snpmap.asc.ohio-state.edu/). Manual interrogation of 100 variants using the Integrated Genome Viewer (61) confirmed the variant presence and call quality metrics in all 100. Manual interrogation of SNP-indel pairs in the pileup confirmed 98% (49 of 50) are valid using the same criteria.

To adjust for mutations in homozygous regions that might have occurred but were then lost due to LOH, the following calculation was used for each homozygous region: {[(homozygous SNPs/all SNPs) + 1] * homozygous SNPs}/length. This effectively doubles the number of homozygous SNPs in hom regions to account for heterozygous SNPs that are lost due to LOH. Even with this doubling, significantly more emergent SNPs occurred in het regions than in hom regions.

### Phylogenetic construction of strain relatedness.

The methods used to construct the phylogeny of the sequenced strains were previously described by Hirakawa et al. (27). Briefly, a representation of the phylogenetic relatedness of the strains was constructed using all whole-genome SNP calls, which totaled 113,339. A distance-based tree was estimated relying on maximum parsimony and a stepwise matrix where homozygous positions are two steps away compared to one step for heterozygous positions. SNP positions were resampled in 1,000 bootstrapped samples, and each node indicates the bootstrap support.

### Determination of LOH.

Previously defined heterozygous and homozygous genomic regions were used as detailed previously (27). Briefly, the SNP density of homozygous and heterozygous positions was calculated across the genome in nonoverlapping 5-kb windows for each isolate. The resulting 5-kb windows were managed as follows: (i) homozygous regions shared between individual strains and SC5314 were identified and marked as homozygous; (ii) a single 5-kb window adjacent to these regions lacking any polymorphisms was merged into homozygous tracts (if present); (iii) contiguous, adjacent windows with a significantly higher frequency of homozygous SNPs than SC5314 homozygous regions (>0.4 SNPs/kb) were merged, allowing one intervening window lacking sufficient polymorphism into homozygous tracts. These regions were defined as homozygous whereas the remaining regions covered by 5-kb windows of the genome were designated heterozygous and contained significantly more heterozygous SNPs. The borders between homozygous and heterozygous regions were manually inspected for accuracy.

### Introgression/tree violations.

Two independent methods were used to assign clade designations for nuclear and mitochondrial genomes in nonoverlapping 25,000-bp or 750-bp windows, respectively. The first process assigned the clade that most closely corresponded to the SNP pattern of the query strain. However, clade I contains fewer SNP calls due to alignment to the SC5314 reference genome (also a clade I isolate), which can introduce an artificial bias. Therefore, a data frame was constructed where each row represented any SNP contained within any strain in the query window. A SNP could be counted only as a single row, so the identity of that SNP position was recorded as “0” for the absence of the SNP, “1” for a heterozygous position, and “2” for a homozygous position. The correlations between the target strain’s resulting numeric SNP profile and that of each of the other strains were individually calculated. Scores from strains within the same clade were averaged as follows and taken as the absolute value: *clade score = abs*(*mean*(*cor*(*query strain SNP*, *strain X SNP*))). The clade with the highest similarity score (and thus the greatest proportion of shared SNPs) was selected as the most similar clade for that window. This process was repeated across the full genome. As a follow-up, this process was repeated by removing all strains within the query strain’s clade and the scores were recalculated.

The second phylogenetic approach used an expression matrix listing the 21 strains against all possible SNP positions present for each nonoverlapping window. For each cell, we assigned a value of 0 if the respective SNP was not present in the respective strain, a value of 1 if one copy of the SNP was present, and a value of 2 if both copies were present. A distance matrix was constructed from these data using a binary method (R dist), finally resulting in a phylogenetic tree (R hclust). The appropriate number of K clusters for the phylogenetic output of each window was estimated by traversing all 21 possible values (1 to 21) incrementally using R cutree. The first k-value that was chosen was the value that allowed the target strain to cluster with at least two other strains that were members of the same clade as defined by the current phylogenetic relationship among the sequenced strains. These criteria effectively eliminated clade E because it contains only one strain. Additionally, this approach was assessed manually for congruence across candidate regions.

### Testing for admixtures.

The NGSAdmix tool was used to perform measurements of genetic admixtures in the sequenced clinical isolates (62). The SNP table for each strain mapped to SC5314 was the input, and the data were compared to clade profiles produced using the three representative strains from each clade highlighted in the text for other comparisons as follows: for clade I, strains 12 C, L26, and P78048; for clade II, strains P57072, P76055, and P76067; for clade III, P34048, P78042, and P57055; for clade SA, strains P87, GC75, and P75063. The data were framed for four K-clusters and visualized.

### Sanger sequencing.

The association between the SNPs and indels identified in the next-generation-sequencing (NGS) data was tested by amplification of specific regions that were either shared among a number of strains (P75063) or strain specific (P60002). Two regions were PCR amplified using primers 5′ AGTCGGTGATGTCTATAGTG 3′ and 5′ GCTGTCCTTGGATCATTGAT 3′ to amplify Chr7:48017.48664 in P75063 and 5′ TTCTGCTGTTGCTGCTGCTA 3′/5′ CTGTCAACTGTCAACCAAAG 3′ to amplify ChrR: 19979596.1998145 in P60002. Amplicons were purified and subjected to Sanger sequencing.

Mitochondrial SNPs were verified using 3 sets of primers to amplify different regions of ChrM across the 21 natural isolates. Two isolates were analyzed from each clade, including strains P60002, P75010, and P94015. Primers 5′ TTAGTAGTGTCGGTGTCTTC 3′ and 5′ AGAGAGGGTTTTGGTTAGGG 3′ were used to PCR amplify ChrM: 4899.6076, 5′ GAATCTCAGAGACTACACGT 3′/5′ GTGGTATACGACGAGGCATT 3′ were used for ChrM: 18265.20660, and 5′ TGGGAAGTAGAGGCTGAAGA 3′/5′ AGGGGCATTATAAGGAGGAG 3′ were used for ChrM: 28094.29548. PCR amplicons were purified and subjected to Sanger sequencing.

### Statistical testing.

Statistical analyses were performed using Student’s *t* test unless otherwise indicated. All statistical tests were performed in the R 3.2.5 programming environment (63).

### Accession number(s).

The genome sequences used in this study are available under BioProject identifier (ID) PRJNA193498 (https://www.ncbi.nlm.nih.gov/bioproject). SNP data are available from dbSNP (https://www.ncbi.nlm.nih.gov/projects/SNP/) under noninclusive accession no. 1456786277 to 1457237021.

10.1128/mBio.01205-18.1 TEXT S1 Supplemental results. Download Text S1, PDF file, 0.3 MB. Copyright © 2018 Wang et al. 2018 Wang et al. This content is distributed under the terms of the Creative Commons Attribution 4.0 International license.
