# Supplementary material for: The Genome of the Human Pathogen Candida albicans Is Shaped by Mutation and Cryptic Sexual Recombination
Source: mBio. 2018 Sep 18;9(5):e01205-18. doi: 10.1128/mBio.01205-18 (PMC6143739; doi:10.1128/mBio.01205-18)
Supplement: TABLE S1 [file mbo004184065st1.pdf]

Table S1. Percentage of the genome encoding SNPs among the sequenced *C. albicans* isolates.

	GC75	P75016	P75063	P87	P60002	P94015	P34048	P78042	P57055	P57072	P76055	P76067	P75010	19F	L26	P37039	12C	P37005	P37037	P78048	SC5314
GC75																					
P75016	0.893741																				
P75063	0.9007	0.890765																			
P87	0.894931	1.13294	1.117622																		
P60002	1.110222	1.083192	1.06102	1.115025																	
P94015	1.135776	0.987413	0.956084	0.978823	0.937475																
P34048	1.153768	1.149679	1.13336	1.124077	1.139423	1.003641															
P78042	1.146375	1.137617	1.11282	0.905635	1.11081	0.988764	0.917376														
P57055	1.1455	1.132947	1.117622	0.817165	1.115032	0.97883	0.912223	0.905642													
P57072	1.136476	1.116866	1.10243	1.11891	1.090627	0.963246	1.141572	1.11751	1.118917												
P76055	1.132506	1.125078	1.101282	1.113072	1.078711	0.951309	1.135643	1.106491	1.113079	0.892411											
P76067	1.129678	1.122649	1.101457	1.114304	1.083087	0.961481	1.136798	1.112449	1.114311	0.903052	0.903276										
P75010	1.102304	1.085306	1.066404	1.086622	1.046787	0.919175	1.106309	1.085502	1.086622	1.084123	1.083024	1.09237									
19F	0.965304	0.952282	0.924489	0.918713	0.921877	0.934605	0.921534	0.956322	0.949636	0.944903	0.933541	0.94106	0.888217								
L26	0.901743	0.960151	0.93247	1.003403	0.930369	0.665127	0.978095	0.964583	0.957428	0.951498	0.940066	0.947137	0.897332	0.501327							
P37039	0.896058	0.955643	0.927212	0.847409	0.92208	0.663909	0.906678	0.960102	0.952086	0.948411	0.936362	0.9412	0.891928	0.501544	0.506536						
12C	1.050092	1.037168	1.007365	1.002388	1.000875	1.012847	1.055923	1.042391	1.033906	1.02799	1.016936	1.022655	0.971437	0.440643	0.600501	0.591631					
P37005	0.897024	0.959059	0.931476	0.849005	0.928948	0.665659	0.908415	0.964163	0.956665	0.950616	0.939296	0.9461	0.896562	0.50391	0.508671	0.507418	0.590483				
P37037	0.898816	0.969651	0.942418	0.850153	0.93945	0.675068	0.909178	0.973817	0.967362	0.961887	0.950525	0.957085	0.90859	0.505177	0.511597	0.520775	0.597309	0.509154			
P78048	0.898025	0.885578	0.849551	0.880026	0.83849	0.666751	0.907862	0.888511	0.880026	0.865681	0.849824	0.862328	0.815632	0.500165	0.508664	0.507341	0.595139	0.508321	0.517709		
SC5314	0.837055	0.824754	0.784653	0.817165	0.767452	0.770343	0.845862	0.828058	0.817172	0.798963	0.782861	0.796176	0.745791	0.472994	0.482221	0.474702	0.537773	0.479687	0.491589	0.506381	
