# Supplementary figures and images for: The Genome of the Human Pathogen Candida albicans Is Shaped by Mutation and Cryptic Sexual Recombination
Source: mBio. 2018 Sep 18;9(5):e01205-18. doi: 10.1128/mBio.01205-18 (PMC6143739; doi:10.1128/mBio.01205-18)

A

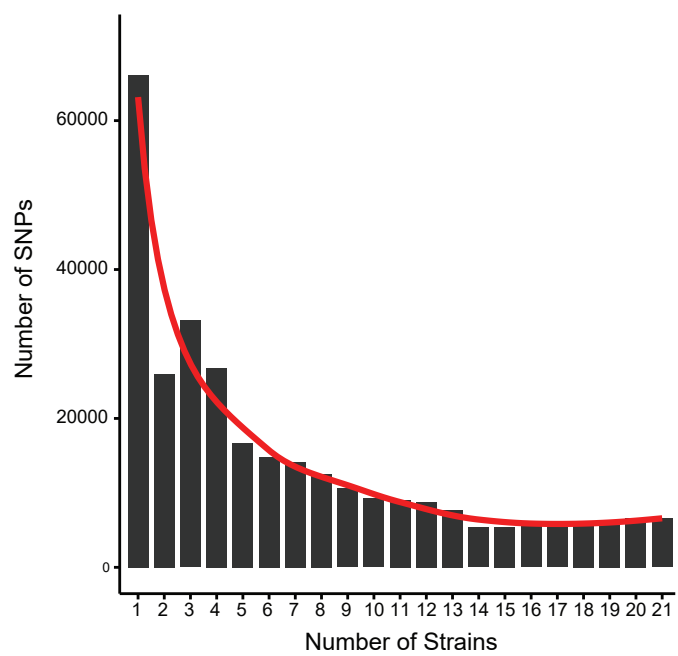

B

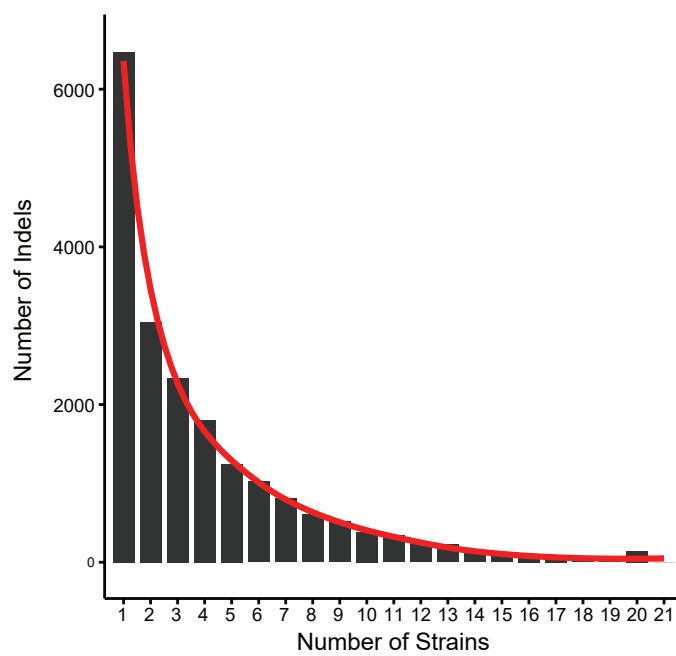

Supplement: FIG S1 [file mbo004184065sf1.pdf]

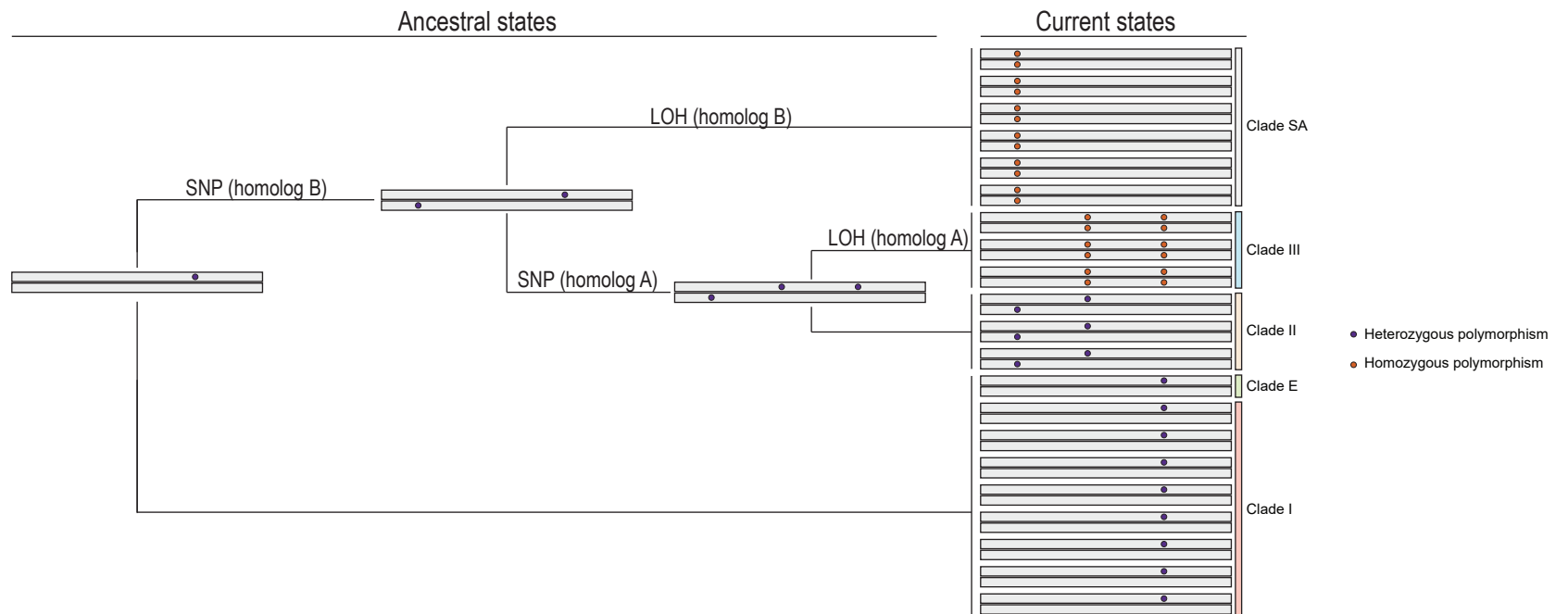

Supplement: FIG S2 [file mbo004184065sf2.pdf]

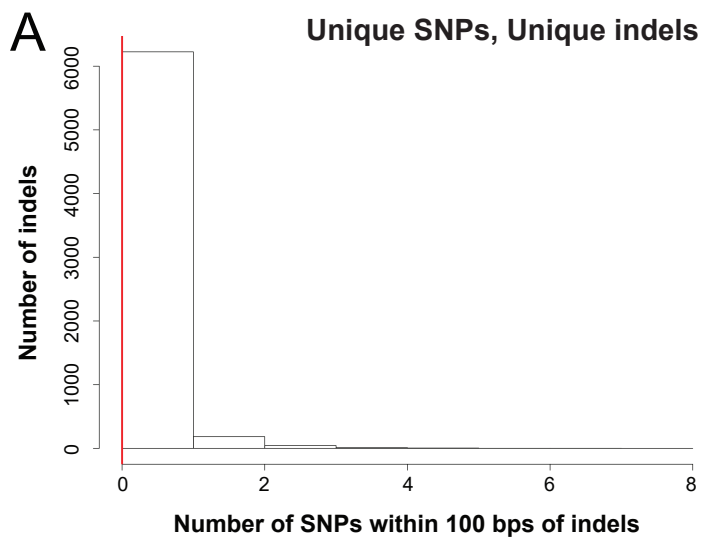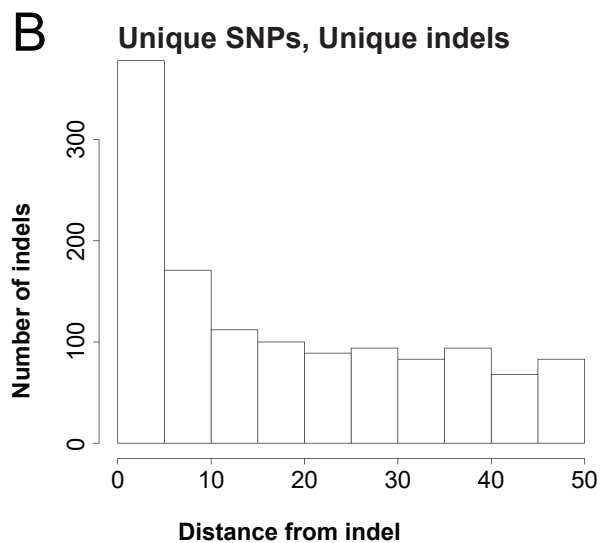

Supplement: FIG S3 [file mbo004184065sf3.pdf]
