# Supplementary material for: The Genome of the Human Pathogen Candida albicans Is Shaped by Mutation and Cryptic Sexual Recombination
Source: mBio. 2018 Sep 18;9(5):e01205-18. doi: 10.1128/mBio.01205-18 (PMC6143739; doi:10.1128/mBio.01205-18)
Supplement: TEXT S1 [file mbo004184065s1.pdf]

SUPPORTING INFORMATION

SI Results

Base substitutions in *C. albicans*

As expected, the number of strain-specific mutations increased with longer branch lengths from the nearest node in the phylogenetic tree (SNPs, $r_s = 0.60$, p = 4.2E-3; indels, $r_s =$ 0.42, p = 0.055; Fig. 1B and Fig. S13). Correlation between these metrics of strain identity supports the use of strain-specific mutations in assessing mutational patterns.

In many eukaryotes, base-substitution mutations are biased towards transitions over transversions, although the cause of this bias is not completely clear [33]. In *C. albicans*, base substitutions also favored transitions over transversions for both strain-specific SNPs and total SNPs, χ^2 ((11, N = 66086) = 18182, p < 2E-16 and (11, N = 302641) = 628000, p < 2E-16, respectively). The ratio of transitions to transversions was 2.21 for strain-specific SNPs and 2.50 for all SNPs (Fig. S14). Both coding and noncoding regions encoded more strain-specific transitions than transversions, although coding sequences were more biased than noncoding regions (2.74 versus 1.80, respectively). Base substitutions displayed a 1.39-fold bias towards introducing A/T instead of G/C for strain-specific SNPs that shrank to 1.03-fold when including all SNPs. The fact that substitutions favor transitions resulting in A/T suggests that this may contribute to the overall A/T richness of the *C. albicans* genome [31].

Analysis of the global distribution of strain-specific SNPs revealed a bias against the accumulation of these mutations within protein-coding genes. Thus, most strain-specific polymorphisms (33,818 of 66,086 SNPs and 5,502 of 6,474 indels) were present within the 36.7% of the genome representing intergenic regions, suggesting that mutations in coding sequences are selected against (p = 9.71E-16; Fig. S15A,B). As a result, relatively few strain-specific SNPs were present within ORFs across the twenty-one sequenced strains (Fig. S15C).

We found that 259 genes exhibited significantly greater SNP densities per nucleotide (nt) than the 0.004 SNPs/nt average for all *C. albicans* ORFs (Fig. S15C, Table S6). SNP densities within enriched genes were equal to or greater than the intergenic average (0.0066 vs. 0.0063, respectively). Protein-coding genes within this group lacked any enrichment for gene ontology (GO) annotations or pathways (Table S6). However, noncoding snoRNAs (small nucleolar RNAs) were significantly overrepresented among 'faster-evolving genes' by GO term analysis, χ^2 ((2, N = 5) = 15.6, p = 7.90E-5; Fig. S15D). The five snoRNAs identified from GO enrichment had mutation rates greater than 0.02 SNPs/nt, significantly higher than that of the average rate of 0.0063 SNPs/nt within intergenic regions. Strain-specific polymorphisms clustered towards the 5' end of the snoRNAs (Fig. S15E) and could contribute to variation in functional aspects of protein translation, although this possibility was not explored here.

Indels in *C. albicans*

An inspection of strain-specific indels revealed that 3527 (54.5%) were deletions and 2948 (45.5%) were insertions. Indels ranged in size from 1 bp to 10 bp with the majority of longer events being insertions (Fig. S16). The frequency of both insertions and deletions decreased as mutations became larger, suggesting that smaller events occur more frequently or are less detrimental to the cell and therefore are retained more often. The incidence of ±3 nucleotide indels (21.9% of the total) was higher than that expected by chance. When indels were separated into genic or intergenic mutations, intergenic mutations followed a Poisson distribution centered on 0, whereas genic mutations were vastly overrepresented for ±3 nucleotide indels that do not shift the reading frame (Fig. S16). Only ~15% of all indels fell within ORFs ($p < 2.2E-16$) suggesting that, as with SNPs, indels are selected against within coding sequences (Fig. S15B).

Indels have been commonly associated with specific genomic features such as repetitive sequences in other species [34, 35]. Across the sequenced *C. albicans* isolates, there was a

total of 19,581 indel sites across the genome. Of these, 465 indel sites (2.37%) were located within annotated repetitive sequences (long terminal repeats (LTRs), major repeat sequences (MRSs), and retrotransposons). Total indels are therefore overrepresented within these repetitive features (two-tailed Brunner-Munzel (BM) test = 5.15, df = 182.05, p = 6.65E-7). Likewise, strain-specific indels were significantly enriched within repetitive features (47 of 6475; BM test = 13.004, df=182, p<2.2E-16). Both total and strain-specific SNPs also clustered within repetitive elements (BM test = 14.98, df = 315.62, p < 2.2E-16 and BM test = 12.26, df = 240.02, p < 2.2E-16, respectively). Thus, mutations within the *C. albicans* genome are enriched within repetitive regions similar to what has been observed in other species [34, 35].

Association of SNPs and indels

Similar to our analysis of strain-specific SNPs and indels described in the main text, an examination of all annotated SNPs and indels found them to be associated with each other in *C. albicans* genomes (Fig. S17A). Again, all SNPs were significantly enriched within a 100 bp window surrounding each indel (Wilcoxon test (W(1.66E11)), p<2.2E-16, Fig. S17B). These polymorphisms clustered within the proximal 10 bp to each indel but did not overlap the indel position (Fig. S17C), similar to strain-specific mutations. Thus, SNPS and indels display the same tight association with one another regardless of whether these are strain-specific or total polymorphisms.

To assess the accuracy of regions containing indels and adjacent SNPs, 21 regions were Sanger sequenced across the indel-SNP associations. Sanger sequencing confirmed 17 of 21 associations (81%) identified from variant calling of the assembled 21 genomes. These validated regions contained identical variants at the predicted position and the calculated allele frequencies as that produced from genome assembly (Fig. S4). Importantly, the interrogated regions represent highly repetitive stretches that are more likely to be called incorrectly by whole

genome analysis. Thus, the high accuracy of variant calls among the Sanger sequenced positions likely reflects that most variant positions are accurately called.

In some species, the introduction of indels can influence the observed mutational bias towards either transitions or transversions [35, 36]. To address this possibility in *C. albicans*, the transition:transversion ratio was determined for the ~500 strain-specific SNPs located within 10 bp of strain-specific indels. Although base substitutions still slightly favored transitions, the 1.17 transition:transversion ratio was significantly lower than the genome-wide average ratio of 2.21 ($p = 5.87E\text{-}7$). This is consistent with mutations close to indels exhibiting a reduced bias towards transitions over transversions due to recruitment of error-prone polymerases during DNA repair [35]. We therefore suggest that a similar mechanism operates in *C. albicans* and can account for the increased mutation rate adjacent to indels, as well as the local bias in the transition:transversion ratio.

Loss of Heterozygosity tracts in *C. albicans*

Mapping the LOH regions among the sequenced *C. albicans* isolates revealed that out of a total of 336 chromosome arms in the 21 isolates, 155 of these arms show evidence of having undergone a long-tract LOH event. Thus, almost half of the chromosomes contained an LOH of greater than 50 kb. LOH frequency decreased towards the centromeres and did not occur across centromeres except during LOH of whole chromosomes (Fig. S18A).

Association of mutations with LOH

The previous study by Hirakawa *et al*. identified extensive loss of heterozygosity (LOH) tracts in the 21 sequenced *C. albicans* isolates [28]. Consequently, LOH breakpoints were mapped in each isolate and emphasis was placed on the distribution of LOH events around the mating type-like (*MTL*) locus on Chr5. The current study extends the analysis of LOH patterns in *C. albicans* genomes by determining if genome-wide patterns of LOH exist, and if there is an

association between LOH tracts and other mutational classes such as base substitutions or indels. Importantly, aneuploidy did not significantly alter the frequency of heterozygous and homozygous intervals along aneuploid chromosomes relative to euploid chromosomes ($p = 0.756$).

We note that heterozygous SNPs may have arisen in hom regions but, in some cases, been eliminated by a subsequent LOH event. As LOH can occur in one of two possible directions (due to loss of either homolog A or homolog B), we accounted for mutations potentially lost via LOH by doubling the number of homozygous, strain-specific SNPs within hom regions. Even with this adjustment, het regions still contained a greater density of strain-specific SNPs than hom regions (two-sided BM test = -8.74, df = 717.58, $p<2.2E-16$). The ~2-fold greater accumulation of polymorphisms in het over hom regions of the *C. albicans* genome shows parallels with the ~3.5-fold higher mutation rate observed in het vs. hom regions of the *Arabidopsis* genome during meiosis [37].

Sites close to recombination events, including LOH events, have been shown to be associated with elevated mutation rates in some species [36, 38-40]. To determine if there is an increased frequency of SNPs in regions proximal to LOH tracts in *C. albicans*, the density of SNPs at heterozygous-homozygous transition points was investigated. Analysis of the 745 identified transition regions included 1 kb of DNA on either side of the junction points between het and hom regions (with the latter inferred to represent LOH tracts). The SNP density within these transition regions was significantly lower than that in the rest of the *C. albicans* genome (one-sided BM test = -35.415, df = 748.8, $p <2.2E-16$; Fig. S18B). Furthermore, SNP density was similar on both het and hom sides of the LOH breakpoint. Thus, base substitutions appear to accumulate less frequently in regions proximal to het/hom breakpoints in the *C. albicans* genome. One caveat noted here is that this result may be influenced by difficulty in the identification of precise breakpoints between het and hom regions of the genome.

When evaluating all polymorphisms, heterozygous regions of the genome contained significantly higher frequencies of SNPs relative to hom regions (1.4E-4 vs. 7.3E-5 SNPs/bp, respectively; two-tailed Brunner-Munzel (BM) test = -10.6, df=786.6, p<2E-16). Therefore, as with the elevated frequency of strain-specific SNPs in heterozygous regions, we find higher frequencies of all SNPs in het than hom regions, indicating that this pattern has been retained during species evolution.

The association of indels with heterozygous and homozygous regions of the genome was more complex. Strain-specific indels were more commonly found in hom regions than in het regions (BM test = -3.22, df=878.4, p=0.0013). In contrast, analysis of all indels displayed a highly significant enrichment in het regions compared to hom regions (BM test = -5.78, df=729.8, p=1.08E-8). Thus, although strain-specific indels are more commonly found in hom regions, het regions encode significantly more indels overall, suggesting that a simple relationship between indel formation/retention and het/hom regions does not exist.

LOH regions were defined by 5 kb windows encoding >0.4 events per kb. We note here that this classification could misclassify some ‘hom’ regions as ‘het’ regions and vice versa. However, misclassification of a subset of these regions would likely result in our analysis underestimating any divergent mutational trends between het and hom regions.

Impact of phylogenetic branch length on evolutionary trends

Phylogenetic reconstruction produced longer individual branch lengths for three strains, P60002, P75010, and P94015, compared to the other 18 strains in this study (Fig. 1A). To investigate if the described evolutionary associations between mutations are biased by the inclusion of these three ‘outlier’ strains, or by having unequal numbers of strains representing each clade, three strains were picked from each of the four major clades (I: 12C, L26, P78048; II: P57072, P76055, P76067; III: P34048, P78042, P57055; SA: P87, GC75, P75063) and the

mutational trends reassessed. In total, these 12 strains accounted for 53% of all strain-specific SNPs and 25% of all strain-specific indels, leaving 31,090 strain-specific SNPs and 4,947 strain-specific indels, respectively.

The association between SNP and indel polymorphisms was still observed when analyzing the more limited set of 12 strains, as we again found a strong association between the positions of strain-specific SNPs and indels (Wilcoxon test (W(3.04E7)), p<2.2E-16, Fig. S5A,B). Similarly, analysis of the association between all SNPs and indels revealed a tight association across the 12 strains (Wilcoxon test (W(2.12E7)), p<2.2E-16, Fig. S5C). Thus, the association between SNPs and indels across the genome was robust across *C. albicans* isolates.

We next examined whether the overrepresentation of polymorphisms accumulating in heterozygous regions of the genome was still observed within the set of 12 strains. Strain-specific polymorphisms were still more prevalent in het than hom regions of the genome when analyzing polymorphisms from this set (SNPs; BM test = --7.558, df=377.0, p<3.14E-13, indels; BM test = -2.35, df=379.7, p=1.97E-2, Fig; S19A,B). This association also existed among all polymorphisms, as polymorphisms were overrepresented in heterozygous regions of the genome in the 12 strains (SNPs; BM test = -14.80, df=422.6, p<2.2E-16, indels; BM test = -3.188, df=321.8, p=1.57E-3).

Finally, we identified genes enriched for strain-specific base substitutions among this set of 12 strains. A total of 15 genes encoded greater than 0.02 SNPs/nt (Figure S19C). As observed for all strain-specific SNPs, snoRNAs were greatly overrepresented among genes for strain-specific polymorphisms (Fig. S19D) and these mutations clustered towards the 5' end of the snoRNA sequence (Fig. S19E). In contrast, no GO terms were significantly enriched among genes with high numbers of all base substitutions among the 12 strains. Therefore, analysis of the more limited set of 12 strains representing all 4 major clades did not significantly alter the association of mutation type, the accumulation of SNPs in heterozygous regions, or the class of

functional RNAs most frequently containing emergent base substitutions despite the loss of the majority of strain-specific polymorphisms relative to the set of 21 isolates.

Genome evolution processes contributing to SNP incongruence

The plasticity of the *C. albicans* genome allows it to adapt quickly through a combination of mechanisms that potentially include both LOH and mating (4-6). The ability to decipher between these two possibilities is challenging in heterozygous diploid genomes where different patterns of LOH can produce genomes that appear 'recombinant' despite a clonal origin. For example, consider a strain encoding heterozygous SNPs along both chromosome homologs. Homozygosis of one homolog or the other will generate strains that have different SNP patterns relative to each another, although both will share SNPs with the parental strain that did not undergo homozygosis (Fig. S9). In isolation, the two homozygosed strains would be viewed as unrelated to one another because of their completely different SNP patterns despite sharing a common origin. If the parental strain was also present, it would appear to be the 'recombinant' product of mating between the two homozygosed strains (Fig. S9). Additionally, short-tract LOH events could further complicate analysis due to shuffling of the polymorphic patterns between related strains (Fig. S9).

Careful analysis of the 21 genome sequences found examples where 'identity by descent' patterns have been broken by different LOH events occurring in different lineages. One example involves *orf19.4819* which is heterozygous for four SNPs in Clade II (Fig. 2D,E). Most Clade I strains have retained only one of the four SNPs, whereas Clade III isolates and the Clade E isolate have retained the other three SNPs. Given the position of these strains in the phylogenetic tree, the most parsimonious explanation is that Clade II has retained the ancestral pattern of heterozygous SNPs for *orf19.4819*, and that the other lineages underwent loss of one homolog or the other generating the extant patterns shown. A second example is shown for SNPs in *orf19.6605* in which it appears that extant strains lost one homolog or the other,

followed by additional mutagenic events (Fig. S8A,B). These patterns are mostly easily recognized when all three SNP configurations are present in a population: one isolate with the parental configuration of heterozygous SNPs, one isolate in which LOH has homozygosed the SNPs for one allele, and another isolate in which SNPs have homozygosed for the other allele (Fig. 2D,E).

Mating between strains is a second key mechanism that can distort mutation patterns from that expected by 'identity by descent'. A possible indication of mating is a sudden break of homology along a chromosome so that SNPs switch from matching one clade to matching a second clade (Fig. 3A,B,D). We also found evidence of mating by identifying strains that have inherited one chromosome homolog from two different extant strains (Fig. 3C, S9). The genotype of the resulting strain will be a composite of polymorphisms from each parent, although additional recombination or mutation events can make such regions challenging to identify. Inference of these events also requires phasing of SNPs for each homolog in both parental strains, as we performed for the region on Chr3 in Fig. 3C. In this case, we reason that it is difficult to envisage how this pattern of SNPs could be generated by LOH events, as the SNPs reflect the expected pattern of heterozygous and homozygous positions when the two parental homologs are combined. Furthermore, we emphasize that the SNPs involved are identical (both in their positions and in the actual nucleotide substitutions) in the highlighted regions. The most parsimonious explanation is that this section of the P94015 genome therefore arose via a hybridization event between a Clade III strain and a Clade SA strain.

REFERENCES

1. Rosenberg MS, Subramanian S, & Kumar S (2003) Patterns of transitional mutation biases within and among mammalian genomes. *Mol Biol Evol* 20(6):988-993.
2. Butler G*, et al.* (2009) Evolution of pathogenicity and sexual reproduction in eight *Candida* genomes. *Nature* 459(7247):657-662.
3. Jovelin R & Cutter AD (2013) Fine-scale signatures of molecular evolution reconcile models of indel-associated mutation. *Genome Biol Evol* 5(5):978-986.
4. Zhang N*, et al.* (2015) Selective advantages of a parasexual cycle for the yeast *Candida albicans*. *Genetics* 200(4):1117-1132.
5. Bennett RJ, Forche A, & Berman J (2014) Rapid mechanisms for generating genome diversity: whole ploidy shifts, aneuploidy, and loss of heterozygosity. *Cold Spring Harb Perspect Med* 4(10).
6. Ciudad T, Hickman M, Bellido A, Berman J, & Larriba G (2016) Phenotypic consequences of a spontaneous loss of heterozygosity in a common laboratory strain of *Candida albicans*. *Genetics* 203(3):1161-1176.
